# Mortality in Patients on Renal Replacement Therapy and Permanent Cardiac Pacemakers

**DOI:** 10.1155/2014/284172

**Published:** 2014-05-26

**Authors:** Gabriel Vanerio, Cristina García, Carlota González, Alejandro Ferreiro

**Affiliations:** ^1^CASMU Arrhythmia Service, 8 de Octubre 3310, 11600 Montevideo, Uruguay; ^2^British Hospital, Avenida Italia 2420, 11600 Montevideo, Uruguay; ^3^Uruguayan Registry of Dialysis, Uruguay; ^4^Nephrology Clinic, Hospital de Clinicas, Faculty of Medicine, The University of the Republic, Avenida Italia s/n, 11600 Montevideo, Uruguay

## Abstract

End stage renal disease is a relatively frequent disease with high mortality due to cardiac causes. Permanent pacemaker (PM) implantation rates are also very common; thus combination of both conditions is not unusual. We hypothesized that patients with chronic kidney disease with a PM would have significantly higher mortality rates compared with end stage renal disease patients without PM. Our objectives were to analyze mortality of patients on renal replacement therapy with PM. 2778 patients were on renal replacement therapy (RRT) and 110 had a PM implanted during the study period. To reduce the confounding effects of covariates, a propensity-matched score was performed. 52 PM patients and 208 non-PM matched patients were compared. 41% of the PM were implanted before entering the RRT program and 59% while on RRT. Mortality was higher in the PM group. Cardiovascular disease and infections were the most frequent causes of death. Propensity analysis showed no differences in long-term mortality between groups. We concluded that in patients on RRT and PM mortality rates are higher. Survival curves did not differ from a RRT propensity-matched group. We concluded that the presence of a PM is not an independent mortality risk factor in RRT patients.

## 1. Background

Permanent cardiac pacing (PM) is the treatment of choice in severe and symptomatic bradycardia. Several independent factors such as age, gender, comorbidities, presence of structural heart disease, stimulation modalities, index arrhythmia, and initial symptoms are associated with mortality [1–4].

End-stage renal disease is a relatively common condition associated with high mortality due to cardiovascular causes. Most deaths in patients on renal replacement therapy (RRT) are attributed to sudden cardiac death, which accounts for approximately one-quarter of all deaths [5–9]. Therefore, as chronic kidney disease is an increasingly prevalent condition and an independent risk factor for cardiovascular mortality, the presence of a permanent pacemaker is likely to increase mortality.

To our knowledge, no data is available regarding mortality in patients on RRT with permanent pacemakers (PM) [10–12]. These groups of patients are not included in the current guidelines [13, 14]. Mortality rates are likely to be higher due to the advanced age, the high incidence of stroke, and comorbidities. Estimation of outcomes after PM placement in long-term dialysis patients is needed to evaluate the risks and benefits of permanent cardiac stimulation [5, 6, 15–21].

Our objectives were

1. to analyze mortality in RRT patients with and without a PM;
2. to compare survival and describe baseline characteristics of patients with PM on a RRT program.

## 2. Methods

We performed a longitudinal retrospective study from January 2003 to December 2008. Data was obtained from the Uruguayan National Resource Fund (FNR) database (which includes 99% of PM implants and all dialysis units in Uruguay) [22]. The FNR is a nongovernmental public organization that provides financial coverage of medical procedures for the entire Uruguayan population. The FNR ensures financing and evaluates the quality of care provided to patients, controlling the processes and outcomes of the funded procedures.

Demographic data, comorbidities, time on RRT, clinical conditions, and the functional status evaluated by Karnofsky modified score (4 categories) were registered to perform comparisons between groups [23]. Data on comorbid conditions at the beginning of the study and the evolution were prospectively recorded using a standardized data collection tool (Basic Data and Evolution Questionnaire). We restricted the analysis to the first device implanted. Participants were followed up from PM implant date until March 30, 2010, or death.

### 2.1. Baseline Covariates

For each patient, we obtained demographic data and cause of renal failure from the FNR database. Variables included risk factors for mortality, such as history of myocardial infarction, coronary heart disease, ischemic stroke or transient ischemic attack, congestive heart failure, cardiac arrest, atrial fibrillation, chronic obstructive lung disease, cancer, and diabetes.

### 2.2. Outcomes

Outcomes of interest were all-cause mortality. Mortality date and cause of death were extracted from the FNR database.

### 2.3. Statistical Analysis

Categorical and continuous data are presented as absolute numbers and percentages or mean values and standard deviations, respectively. Variables were compared by the *χ* ^2^ test, Fisher's exact test or Student's *t*-test, Wilcoxon test, and binary logistic regression when appropriate. Survival curves were constructed by the Kaplan-Meier method. Pooled-over strata log rank test or Breslow test was used for comparing the equality of survival distributions for the different levels of the factors. The log-rank test was used to compare curves. Due to the important imbalance of mortality-associated comorbidities between groups, patients who received a PM device while on RRT were propensity-matched accordingly with a logistic regression derived probability of death, adjusted to the confounding effects of covariates (age, sex, comorbidities, Karnofsky-based functional status, and previous time on dialysis). All variables significant at the *P* < 0.2 level were entered into the multivariate model (forward stepwise binary logistic regression analysis) provided they were present in at least 2% of the sample. Variables were entered into the model separately, beginning with the variable having the highest statistically significant score. Variables that significantly improved the fit of the model were retained and forced into subsequent models. Stability of the model was assessed every time a variable was entered. The final step was to search for first-degree interaction. Criteria to include an interaction term were: (1) significant at *P* < 0.05, (2) 1% of the sample had to exhibit the same combination factors, and (3) the combination should be clinically relevant. All patients were propensity-matched accordingly with the derived probability of death, on a 1 PM × 4 non-PM number of patients basis (1 : 4 ratio). 52/65 (80%) PM patients (all on RRT before the device was implanted) and 208 non-PM matched subjects were suitable for comparison. A two-sided alpha level of 0.05 was considered statistically significant.

## 3. Results

From 2003 to 2008, 7129 new PMs were implanted in Uruguay (1188 per year); during this period, 1432 (20%) PM patients died.

During the same time period, 2778 patients were on RRT, with an unadjusted annual mortality rate of 13.4%. Chronic kidney diseases determinant of the loss of renal function were primary glomerulopathies (12.6%), diabetic nephropathy (21.5%), vascular nephropathy (24.4%), obstructive nephropathy (8.2%), tubule-interstitial nephropathy (2.8%), other causes (28.5%), and unknown cause (2%). Mortality was secondary to cardiovascular diseases in 39.7% (95% CI 34–44), infectious diseases in 19.25% (15–23), discontinuation of treatment in 7.9 (2–12), cancer in 8 (6–9), and other causes in 26% (22–28).

110 of the 2778 (3.9%) RRT patients were recipients of a PM, 41% (45/110) before entering the RRT program, and 59% (65/110) while on RRT.

The PM population on RRT corresponded to 1.9% of all PM implants in Uruguay in the observation period.

### 3.1. RRT Population

**Figure 1:**
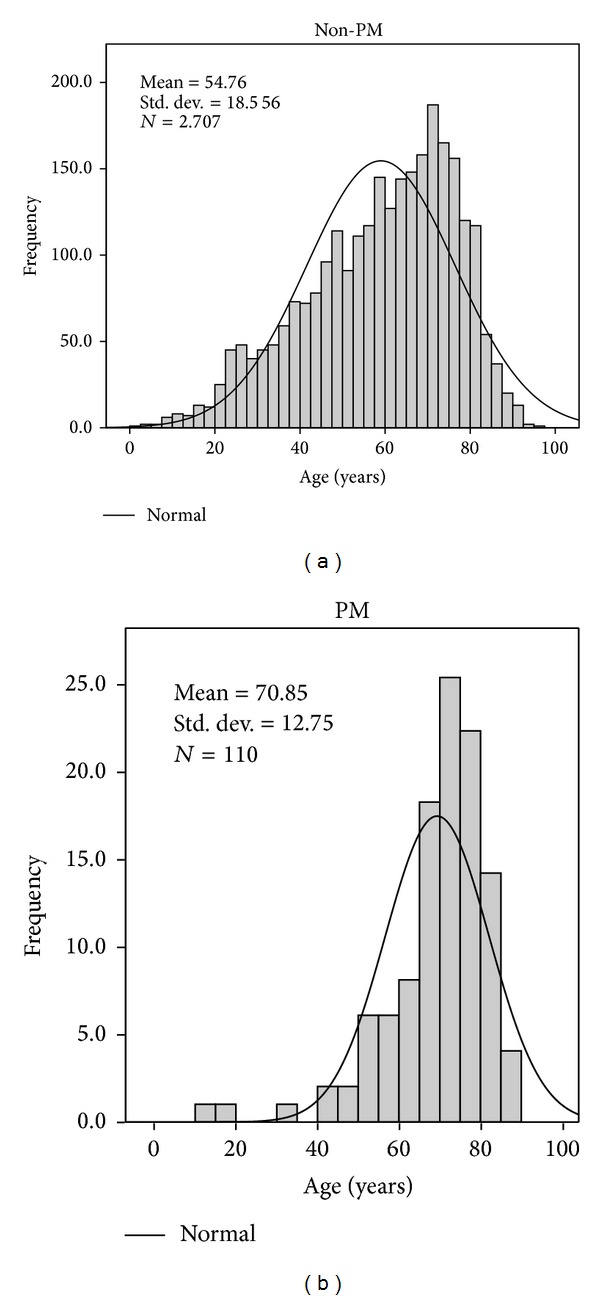
Age histograms from both groups without pacemaker (a) and with pacemaker (b).

Mean followup was 93.6 ± 63 months. Patients in the PM group were older and predominantly male. (Figure 1) Comorbidities were extremely common.  Table 1 shows baseline characteristics and comparison between groups; PM patients were older, with a significant male predominance; Kaplan-Meier survival time was significantly shorter. Within the PM group, there was a slightly higher prevalence of diabetes, and the functional status was significantly lower. A significantly higher incidence of stroke and neoplasic disease was observed. Those who received a PM while on RRT had the highest burden of comorbidities (Table 1).

### 3.2. Study Outcomes

**Figure 2:**
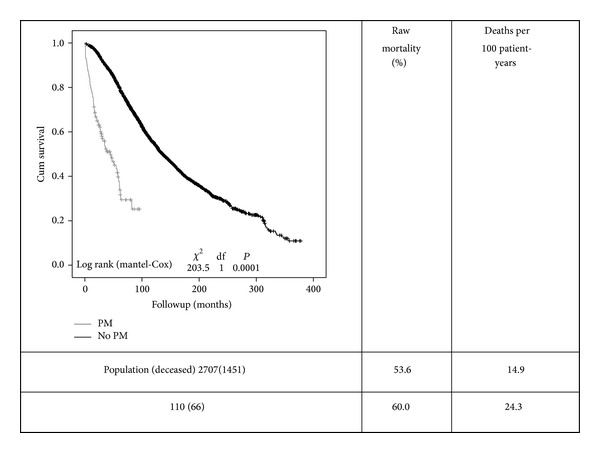
Kaplan-Meier survival curves comparing both groups (PM-pacemaker grey line and non-PM black line). Curves are quite different and show a high mortality in both groups, particularly in the PM group. Mortality (raw and adjusted) rates are shown on the right side of the figure.

Crude all-cause mortality was higher in the pacemaker group (60% versus 54% *P* = 0.2) with a different mean survival time (29.7 ± 2 months versus 96.2 ± 6 months, resp.; *P* < 0.05). The mortality rate was 24.3 versus 14.9 per 100 patient-years, respectively, *P* < 0.05 (Figure 2).

Variables associated with long-term mortality were: odds ratio (95% CI); age 1.053 (1.043–1.062); age > 70 years; 1.5 (1.1–2.0) coronary heart disease; 1.8 (1.4–2.3) diabetes; 2.1 (1.5–2.8) COPD; 2.9 (1.9–4) Karnofsky modified score 1; 1.9 (1.5–2.3) Karnofsky modified score 2; 4.3 (3.09–6) Karnofsky modified score 3; 7.4 (3.3–16) peripheral vascular disease; 3 (2.1–4.3).

### 3.3. Propensity Matched Adjusted Groups

**Figure 3:**
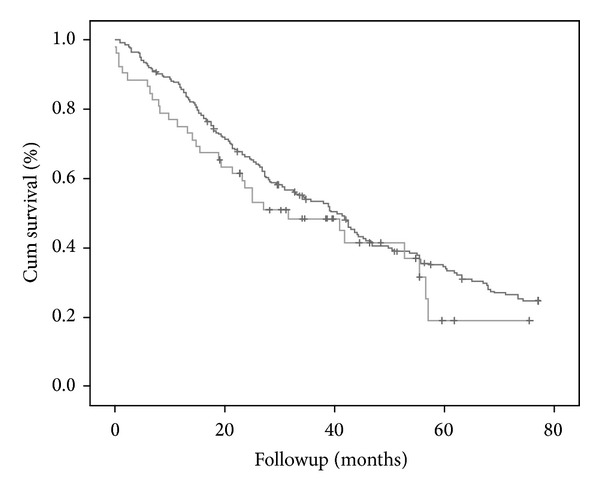
KM survival curve after propensity-matched adjustment. The darker grey line corresponds to the non-PM group and the lighter grey to PM group. There are no differences regarding survival between both groups (*P* = NS).

After propensity adjustment, no differences were observed in long-term mortality between both groups (Table 3 and Figure 3).

Survival curves were not different in the RRT propensity-matched group. Coronary artery disease, age, and the Karnofsky modified score were the only independent variables associated with mortality in the propensity population, without differences between groups.

### 3.4. PM Population

**Figure 4:**
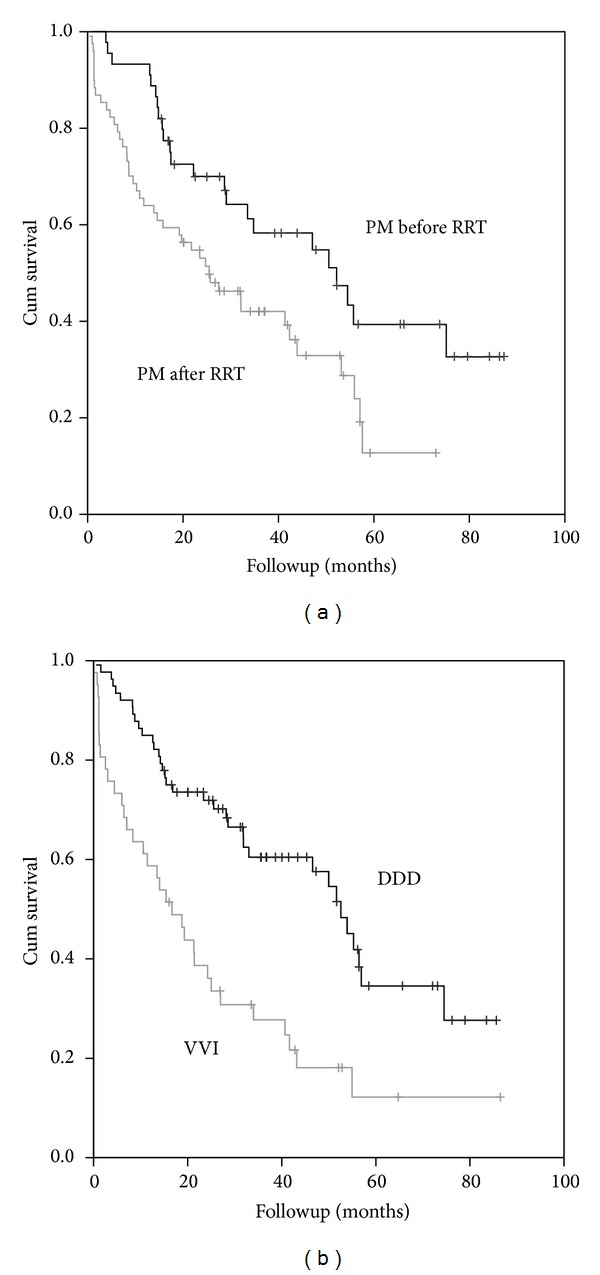
KM survival curves. (a) Mortality related to RRT; the grey lines correspond to the group of PM patients who received the device while on RRT and the black line corresponds to those who received the PM before entering the RRT program. Log rank (mantel-Cox); Chi-Square 6,2 df 1; *P* = 0.012. (b) Mortality regarding PM mode, DDD versus VVI pacing. The grey line corresponds to the VVI PMs and the black line to the DDD devices. Log rank (mantel-Cox) Chi-Square 11,31 df 1; *P* = 0.001.

The PM population could be divided into three groups: 64 (58%) patients who received the PM while on RRT (group A), mean time of 68 ± 56 months on RRT before PM implantation. Group B: 27 (24%) patients received the pacemaker before entering the RRT program (mean time −26 ± 21 months) and Group C: 19 (17%) patients received the device within 3 months before or after entering the RRT program (1 ± 1.35 months). Group A had a significantly higher mortality (65% versus 52% *P* = 0.05, versus group B) and a shorter mean survival time (24 ± 19 versus 36 ± 25 months), with a different age at implantation (68 ± 15 versus 74 ± 7 years, *P* < 0.05) (Figure 4). In these subgroups, the presence of atrial fibrillation was associated with a significantly higher mortality, group A versus group B plus group C, 21% versus 9% (*P* < 0.006). VVI pacing was also associated with higher mortality rates, particularly in group A, 38% (*P* < 0.003) versus 15% in groups B and C together.

Most deaths were cardiovascular (20.9%), and approximately 10% were attributed to cardiac arrest or ventricular arrhythmias (Table 2). Deaths due to infections were common and observed in 18% of the patients, most of them not related to PM infections.

Survival within the PM group was longer in males than females (32 ± 23 versus 23 ± 16 months, *P* < 0.05) despite an older but non-significant age at implantation (male 71.7 ± 11 years versus female 68 ± 14 years). The type of arrhythmia leading to the PM implant had no influence on survival. Patients with a VVI PM had a higher mortality compared to those with dual chamber PMs, with a significantly shorter mean survival time, 16 ± 15 months versus 47 ± 23 months, *P* < 0.05 (Table 4 and Figure 4).

## 4. Discussion

Although PM implant is routinely performed in patients on RRT, there is no relevant data available in this group of patients. Patients on RRT have increased morbidity and mortality and are at increased risk of developing cardiac device-related infections. Interestingly, there is much more information of implantable cardioverter defibrillators (ICDs) and RRT [10, 24–40].

In our study, we found that crude mortality rates were higher in the PM group, averaging 24.3 deaths per 100 patient-years versus 14.9 deaths per 100 patient-years in the RRT population without PM. This finding is probably related to older age and associated comorbidities. However, after propensity adjustment, mortality was similar between groups.

Global mortality rates in patients on RRT and patients without PM were lower when compared to other countries, 18.6 deaths per 100 patient-years of unselected dialysis patients reported in 2008 in the US [5, 41].

Cardiovascular disease and particularly malignant arrhythmias remain the predominant cause of death in RRT patients; however, infections were a very frequent cause of death [31, 32].

Patients with ICDs have much higher mortality rates, with 45 deaths/100 patient-years of followup. Cardiovascular mortality accounts for two-thirds of all deaths, and more than half those deaths were due to arrhythmia despite the type of the implanted device [28, 41, 42].

The high rate of deaths from arrhythmia after ICD-defibrillator or PM placement is consistent with studies showing higher defibrillation thresholds in dialysis patients than in individuals with preserved kidney function [33, 42]. The uremic milieu, the major shifts in potassium and calcium concentrations during hemodialysis [41–44], may increase the likelihood of a sudden increase of capture thresholds, resulting in defibrillation-resistant arrhythmias, bradycardia, heart block, and primary pulseless electrical activity in RRT patients [19, 35].

Although not important in our population as a significant mortality cause, clinicians considering PM therapy should carefully evaluate a history of infections before device placement, an assessment that may be particularly important in patients with catheters or grafts. RRT patients are at great risk of infection due to the repeated exposure during intravenous access creating a permanent potential menace of infection long after PM implantation. Reduction of device-related infections continues to be a clinical challenge. Irrigation of the pocket with antibiotics, antibacterial meshs coated with antibiotics, and/or use of prophylactic antibiotics had been shown to reduce infection rates [45].

Cost-effectiveness analyses should be implemented to assess mortality risk factors in order to clearly define the optimal PM indications in the RRT population, particularly the need for cardiac resynchronization therapy with or without an ICD. Those with depressed left ventricular function and atrial fibrillation have a significantly higher mortality rate.

In the near future, this population will definitively benefit form leadless PM to overcome infections.

### 4.1. Study Limitations

There are several limitations to our study. It was a retrospective observational study and the information contains administrative data rather than clinical records, but the data was prospectively recorded using a standardized data collection instrument, allowing for the best quality of data acquisition in these types of observational studies. The number of patients suitable for the propensity analysis in the PM group was quite small. However, 80% of patients that received a PM while on RRT were included in the propensity risk-adjusted analysis.

Cardiovascular outcomes were not adjudicated independently, and important conditions, device-related complications, potassium level, and medication, were unavailable for analysis.

## 5. Conclusions

Patients on RRT with an implanted PM had significantly higher mortality rates; however, this observation is related to the burden of comorbidity. The presence of a PM is not an independent risk factor for mortality.

Patients on RRT that receive a PM have a worse prognosis than those that had the PM implanted before entering RRT.

Single chamber pacemakers had a significantly higher mortality and should be avoided if possible.

## Figures and Tables

**Table 1:** Patient demographics.

	No PM	PM	PM after RRT (group A + C); see text
*n* (%)	2668 (96)	110 (4)	83 (2.9)
Age (years)	59.0 ± 1	70.8 ± 1*	69.5 ± 14.5**
Female gender *n* (%)	1140 (42)	29 (26)^ŦŦ^	*27 (32.1)^Ŧ^*
Mean survival time (months)	96.2 ± 62	29.7 ± 22*	*24* ± 19**
Previous time in RRT (years)	5.2 ± 4.6	NA	4.8 ± 4.6
Arterial hypertension *n* (%)	1272 (47)	61 (55)	*50 (60) *
SBP > 140 mmHg predialysis *n* (%)	983 (37)	44 (40)	*34 (41) *
Antihypertensive treatment *n* (%)	1042 (38)	53 (48)	*45 (54) *
Hb ≥ 10 g% *n* (%)	609 (22)	8/29 (25)	*5/25 (20) *
Coronary artery disease *n* (%)	569 (22)	35 (32)	42 (51.5)**
Atrial fibrillation *n* (%)	16^§^	35 (32)	*20 (35) *
Valvular heart disease *n* (%)	NA	13 (11.8)	*8 (9) *
Diabetes *n* (%)	575 (21)	26 (23)	23 (27.9)*
(a) Karnofsky 1 *n* (%)	1425 (53)	25 (22)^ŦŦ^	25 (30.9)**
(a) Karnofsky 2 *n* (%)	749 (28)	61 (55)*	31 (38.2)*
(a) Karnofsky 3 *n* (%)	392 (15)	24 (22)	19 (23.5)*
(a) Karnofsky 4 *n* (%)	100 (3.7)	8 (5.4)	6 (7.4)*
Previous stroke *n* (%)	216 (8.3)	24 (22)^Ŧ^	29 (35.3)**
Smoker *n* (%)	347 (13)	18 (16)	21 (27.3)**
Neoplastic disease *n* (%)	202 (7)	19 (17)^ŦŦ^	22 (27.5)^ŦŦ^
Peripheral vascular disease *n* (%)	417 (15)	19 (17)	22 (27.5)**
COPD *n* (%)	212 (7.9)	12 (11)	6 (7.5)
Mortality *n* (mortality/rate 100 patient-years)	1451 (14.9)	66 (24.3)*	29 (35.2)^ŦŦ^

All comparisons versus the non-PM group; *P < 0.05, **P < 0.01, ^Ŧ^ *P* < 0.001, and ^ŦŦ^ *P* < 0.0001.

^§^Data estimated from the international dialysis outcomes and practice patterns study (DOPPS).

NA: not available.

(a) 1: Able to carry on normal activity and to work; no special care needed. 2: Normal activity with effort; some signs or symptoms of disease; unable to work; able to live at home and care for most personal needs; varying amount of assistance needed. 3: Cares for self; unable to carry on normal activity or to do active work; requires occasional assistance but is able to care for most of his personal needs. 4: Unable to care for self; requires equivalent of institutional or hospital care; disease may be progressing rapidly.

**Table 2:** Cause of death in the PM group.

	*N* = 66 (%)
Cardiovascular	17 (20.9)
Cardiac arrest	8 (21.1)
End-stage dilated cardiomyopathy	2 (3)
Myocardial infarction	3 (4.5)
Stroke, including intracranial hemorrhage	3 (4.5)
Pulmonary edema due to exogenous fluid	1 (1.5)
Infection	12 (18.1)
Neoplasic disease	1 (1.5)
Unknown	36 (54.5)

**Table 3:** Characteristics of the propensity-matched adjusted groups.

Variable	No PM (n = 208)	PM (*n*=52)^a^	*P*
Predicted probability	0.64 ± 0.26	0.64 ± 0.25	NS
Age (years)	65.2 ± 13.4	66.9 ± 13.6	NS
Female gender (%)	41.8	42.6	NS
Diabetes (%)	20.1	25	NS
Previous time on RRT (years)	5.06 ± 4.4	5.8 ± 4.6	NS
Coronary heart disease (%)	46.2	49.8	NS
Previous stroke (%)	16.3	23.5	NS
Smoker (%)	11.5	17.3	NS
Neoplastic disease (%)	7.7	15.4	NS
Peripheral vascular disease (%)	21.5	28.8	NS
COPD (%)	6.2	9.6	NS
Functional status			
Karnofsky* 1 (%)	35.9	30.8	NS
Karnofsky* 2 (%)	36.8	40.4	NS
Karnofsky* 3 (%)	21.2	22	NS
Karnofsky* 4 (%)	7.7	5.3	NS

*As in Table 1.

^a^All patients in this group had the PM implanted while on RRT.

**Table 4:** Characteristics of the PM patients by survival outcome.

	Alive	Dead	*P*
*n* (%)	44 (40)	66 (60)	
Age (mean ± SD)	69.7 ± 11	71.6 ± 13	0.3 NS
Female gender *n* (%)	11 (25)	18 (27)	0.8 NS
Followup (months) (mean ± SD)	43 ± 21	21 ± 18	0.05
Arterial hypertension *n* (%)	29 (66)	32 (48)	0.08 NS
Diabetes *n* (%)	4 (9)	10 (15)	0.4 NS
Atrial fibrillation *n* (%)	17 (38)	18 (27)	0.2 NS
Coronary artery disease *n* (%)	6 (13)	13 (19)	0.4 NS
Dilated cardiomyopathy *n* (%)	2 (4)	7 (10)	0.3 NS
LVEF % (mean ± SD)	52.8 ± 2	50.8 ± 1	0.4 NS
Valvular heart disease *n* (%)	3 (7)	10 (15)	0.2 NS
AV block *n* (%)	23 (52)	42 (63)	0.24 NS
Sick sinus syndrome *n* (%)	18 (41)	23 (34)	0.5 NS
VVI PM *n* (%)	8 (18)	32 (48)	0.0013

NS: not significant.
